# Predicting Need for Hospitalization of Patients with Pandemic (H1N1) 2009, Chicago, Illinois, USA

**DOI:** 10.3201/eid1610.091889

**Published:** 2010-10

**Authors:** Shawn Vasoo, Kamaljit Singh, Gordon M. Trenholme

**Affiliations:** Author affiliation: Rush University Medical Center, Chicago, Illinois, USA

**Keywords:** Pandemic (H1N1) 2009, influenza, subtype H1N1, hospitalization, ICU admission, risk factors, viruses, Illinois, dispatch

## Abstract

In the absence of established guidelines for hospitalization of patients with pandemic (H1N1) 2009, we studied emergency department patients to identify clinical parameters that predict need for hospitalization. Independent predictors of hospitalization include multiple high-risk medical conditions, dyspnea, and hypoxia. These findings are easily applicable, with a 79% positive predictive value for hospitalization.

Past influenza outbreaks have shown that limited healthcare resources may be rapidly overwhelmed during an outbreak (*1**,**2*). Guidelines for hospitalization of persons with influenza would help physicians by providing a framework for the initial evaluation and management of patients with influenza. We conducted a study of patients with pandemic (H1N1) 2009 to identify predictors for hospitalization.

## The Study

All patients with confirmed pandemic (H1N1) 2009 infection seen in the emergency department (ED) of Rush University Medical Center (a 613-bed teaching hospital in Chicago) from April 29, 2009, through June 22, 2009, were included in the study. Patients were stratified into 2 groups: hospitalized patients (admitted for at least 24 hours) and nonhospitalized patients (patients discharged from the ED).

Respiratory specimens from ED patients with influenza-like illness were tested by reverse transcription–PCR for respiratory viruses by using the Luminex xTAG RVP (Luminex, Austin, TX, USA), and clinical data were entered into electronic medical records. Specimens positive for nontypeable influenza A by Luminex xTAG RVP were confirmed as pandemic (H1N1) 2009 by using the Centers for Disease Control and Prevention (CDC) reverse transcription–PCR for pandemic (H1N1) 2009 (*3*). Continuous variables that vary with age (respiratory rate, blood pressure, hematologic counts) were regrouped as normal or abnormal by using age-specific normal ranges (*4**,**5*). Obesity (body mass index >30) for adults and children 2–19 years of age and for those with high-risk medical conditions was defined according to CDC guidelines (*6**,**7*).

The Mann-Whitney U test and Pearson χ^2^ test or Fisher exact test were used to compare continuous and categorical variables, respectively. p values <0.05 were considered significant. Backwards stepwise logistic regression was performed for factors associated with hospitalization and intensive care unit (ICU) admission. Goodness-of-fit was determined with the Hosmer-Lemeshow statistic. Data were analyzed by using SPSS version 16.0 (SPSS Inc., Chicago, IL, USA). The study was approved by the institutional review board of Rush University Medical Center.

A total of 189 cases that were identified by review of microbiology records were considered eligible for the study. However, only 83 patients who were examined in the ED were included in the study; the remaining 106 patients were seen at outpatient clinics and private doctors’ offices. Demographic, clinical, and laboratory data of hospitalized patients (32 [39%]) were compared with data from those discharged from the ED (51 patients) (Tables 1, 2).

**Table 1:** Demographics of nonhospitalized and hospitalized patients who had pandemic (H1N1) 2009 infection, Rush University Medical Center, Chicago, Illinois, USA, April 29–June 22, 2009*

Characteristic	Nonhospitalized patients, n = 51	Hospitalized patients, n = 32	ICU patients, n = 16	Nonhospitalized vs. hospitalized patients			ICU vs. non–ICU patients†	
				p value	OR (95% CI)		p value	OR (95% CI)
Median age, y (IQR)	20.0 (9.0–28.0)	12.0 (2.0–38.8)	2.5 (1.13–31.8)	0.70‡	–		0.16‡	–
Age <5 y, no. (%)	8 (15.7)	12 (37.5)	9 (56.3)	0.02§	3.2 (1.1–9.1)		0.002	6.6 (2.0–21.3)
Sex, M/F (% M)	23/28 (45.1)	12/20 (37.5)	5/11 (31.3)	0.50§	0.73 (0.30–1.8)		0.33§	0.56 (0.18–1.8)
Presence of high- risk conditions,¶ no. (%)	21 (41.2)	29 (90.6)	16 (100)	<0.0001§	13.8 (3.7–51.3)		<0.0001	–
No. high-conditions per patient,¶ median (range)	0 (0–2)	2 (0–4)	2 (1–3)	<0.0001‡#	–		<0.0001‡	–
Chronic pulmonary disease, no. (%)	11 (21.6)	13 (40.6)	9 (56.3)	0.06§	2.5 (0.94–6.6)		0.01	4.5 (1.4–14.0)
History of prematurity, no. (%)	0	6 (18.8)	5 (31.3)	0.002	–		0.001	30.0 (3.2–281.8)
Congenital heart disease,** no. (%)	0	2 (6.3)	2 (12.5)	0.15	–		0.04	–
Transplantation,†† no. (%)	1 (2.0)	3 (9.4)	1 (6.3)	0.29	5.2 (0.51–52.1)		1.00	1.4 (0.14–14.6)
Hemoglobinopathy, no. (%)	0	4 (12.5)	2 (12.5)	0.02	–		0.17	4.6 (0.6–35.8)
Diabetes mellitus, no. (%)	3 (5.9)	5 (15.6)	3 (18.8)	0.25	3.0 (0.66–13.4)		0.18	2.9 (0.6–13.5)
Chronic neurologic disease	2 (3.9)	7 (21.9)	4 (25.0)	0.02	6.9 (1.3–35.5)		0.07	4.1 (1.0–17.7)
Immunosuppression, no. (%)	2 (3.9)	5 (15.6)	3 (18.8)	0.10	4.5 (0.82–25.0)		0.13	3.6 (0.7–18.2)
Malignancy, no. (%)	0	3 (9.4)	1 (6.3)	0.054	–		0.48	2.2 (0.2–25.5)
Pregnancy, no. (%)‡‡	1 (4)	3 (15.0)	0	0.29	4.8 (0.46–49.6)		0.56	–

*p values by Fisher exact test except as indicated. ICU, intensive care unit; OR, odds ratio; CI, confidence interval; IQR, interquartile range; –, not applicable. †Nonhospitalized patients + hospitalized patients not in ICU; n = 67. ‡Mann-Whitney U test. §Pearson 2-sided χ^2^ test. ¶High-risk conditions as defined by Centers for Disease Control and Prevention: <5 y or >65 y; pregnancy; immunosuppression; chronic pulmonary, cardiovascular, hepatic, hematologic, neurologic, neuromuscular, or metabolic disorders; long-term aspirin therapy in those <18 y of age. #Significant on multivariate analysis. **Tetralogy of Fallot (1), patent ductus arteriosus status postmedical closure (1). †† Renal transplant (2), liver transplant (1), heart transplant (1). ‡‡Percentage of female patients.

**Table 2:** Clinical characteristics of nonhospitalized and hospitalized patients who had pandemic (H1N1) 2009 infection, Rush University Medical Center, Chicago, Illinois, USA, April 29–June 22, 2009*

Characteristic	Nonhospitalized patients, n = 51	Hospitalized patients, n = 32	ICU patients, n = 16	Nonhospitalized vs. hospitalized patients			ICU vs. non–ICU patients†	
				p value	OR (95% CI)		p value	OR (95% CI)
Duration of ILI before evauulation, d, median (range)	2 (0–7)	3 (1–7)	3 (1–7)	0.15‡	–		0.20‡	–
Subjective fever, no. (%)	46 (90.2)	27 (84.4)	14 (87.5)	0.50	0.59 (0.16–2.2)		1.00	0.95 (0.18–5.0)
Headache, no. (%)	18 (35.3)	5 (15.6)	1 (6.3)	0.05§	0.34 (0.11–1.0)		0.03	0.14 (0.02–1.1)
Cough, no. (%)	50 (98.0)	25/31 (80.7)	14 (87.5)	0.01	0.08 (0.01–0.73)		0.62	0.57 (0.10–3.3)
Rhinorrhea, no. (%)	40 (78.4)	13/31 (41.9)	7 (43.8)	0.001§	0.20 (0.08-0.53)		0.05§	0.34 (0.11–1.0)
Sore throat, no. (%)	24 (47.1)	2/31 (6.5)	1 (6.3)	<0.0001§	0.08 (0.02–0.36)		0.02§	0.11 (0.01–0.88)
Myalgia, no. (%)	21 (41.2)	6/31 (19.4)	2 (12.5)	0.04§	0.34 (0.12–0.98)		0.053§	0.23 (0.05–1.1)
Dyspnea, no. (%)	2 (3.9)	15 (46.9)	11(68.8)	<0.0001§¶	21.6 (4.5–104.4)		<0.0001¶	22.4 (5.8–86.2)
Nausea/vomiting, no. (%)	14 (27.5)	9/31 (29.0)	4 (25.0)	0.88§	1.1 (0.40–2.9)		1.00	0.83 (0.24–2.9)
Obesity (BMI >30 or weight >95th percentile), no. (%)	12/37 (32.4)	11/22 (50.0)	4/10 (40.0)	0.18§	2.1 (0.71–6.2)		1.00	1.1 (0.26–4.2)
Tachypnea, no. (%)	9/49 (18.4)	16/31 (51.6)	10 (62.5)	0.002§	4.7 (1.7–13.0)		0.003§	5.4 (1.7–17.5)
O_2_ saturation, % (range)	99 (96–100)	95 (65–100)	92 (65–100)	<0.0001‡¶	–		<0.0001‡¶	–
Hypoxia (SpO_2_ <92%), no. (%)	0	10 (31.3)	9 (56.3)	<0.0001	–		<0.0001	84.9 (9.3–772.0)
Lymphopenia,# no. (%)	9/12 (75.0)	21 (65.6)	10 (62.5)	0.72	0.64 (0.14–2.80)		0.54§	0.67 (0.18–2.5)
Thrombocytopenia,# no. (%)	1/12 (8)	8 (25.0)	5 (31.3)	0.41	3.70 (0.41–33.00)		0.25	2.7 (0.6–12.2)
Acute renal failure,** no. (%)	0	5 (15.6)	4 (25.0)	0.007	**–**		0.004	22.0 (2.3–214.2)
Infiltrate on chest radiograph, no. (%)	0	11/29 (37.9)	11 (68.9)	0.001	–		<0.0001	–

*p values by Fisher exact test except as indicated. Values given as no./no. indicate number of patients for whom results were available (if less than total no. patients in category). ICU, intensive care unit; OR, odds ratio; CI, confidence interval; ILI, influenza-like illness; –, not applicable; BMI, body mass index; SpO_2_, pulse oximeter oxygen saturation. †Nonhospitalized patients + hospitalized patients not in ICU; n = 67. ‡Mann-Whitney U test. §Pearson 2-sided χ^2^ test. ¶Significant on multivariate analysis. #Lymphopenia <1,500 lymphocytes/mm^3^, thrombocytopenia <150,000 thrombocytes/mm^3^. **Assuming that patients who did not have biochemical testing did not have acute renal failure.

Most patients were African American (63%) or Hispanic (27%); 48 patients (58%) were female. The median age of hospitalized patients was 12 years (interquartile range 2–38.8 years) versus 20 years (interquartile range 9–28 years) for nonhospitalized patients (p = 0.70). Of 32 hospitalized patients, 17 (53%) were children; most (71%) of these children were <5 years of age. The most common admitting diagnoses were pneumonia (11 patients), viral syndrome (5 patients), influenza (4 patients), and asthma exacerbation (4 patients). Univariate analysis showed that being <5 years of age was significantly associated with hospitalization (38% hospitalized vs. 16% nonhospitalized, odds ratio 3.2, 95% confidence interval 1.1–9.1; p = 0.02).

Hospitalized patients were significantly more likely to report a high-risk medical condition than were nonhospitalized patients (p<0.0001). Univariate analysis showed that the following high-risk medical conditions were also significantly associated with hospitalization: history of prematurity, hemoglobinopathy, and chronic neurologic disease (p<0.05). A trend toward a higher incidence of chronic pulmonary disease was seen in hospitalized patients (41% vs. 22% of nonhospitalized patients; p = 0.06). Obesity was not found to be a significant risk factor for hospitalization (p = 0.18).

Patients with dyspnea were significantly more likely to be hospitalized (p<0.0001). Hospitalized patients had lower pulse oximeter oxygen saturation (SpO_2_; median 95%, range 65%–100%) than nonhospitalized patients (median 99%, range 96%–100%; p<0.0001). Tachypnea and hypoxia (SpO_2_ <92%) were significantly associated with hospitalization (p = 0.002 and p<0.0001, respectively). Five of 39 patients with measured creatinine had evidence of acute renal failure, which was significantly associated with hospitalization (p = 0.007). A chest radiograph showed an infiltrate in 11 of 51 patients, and all 11 patients were hospitalized (p = 0.001). Hypoxia was a strong predictor of a chest radiograph finding of infiltrate (odds ratio 50.7, 95% confidence interval 7.2–354.3; p<0.0001). Multivariate analysis showed that high-risk medical conditions (median number of high-risk conditions 2 vs. 0; p = 0.01), dyspnea (p = 0.01), and oxygen saturation (median SpO_2_ 95% vs. 99%; p = 0.004) were found to be significantly associated with hospitalization (Table A1).

Sixteen of 83 (19%) study patients were admitted to an ICU. No deaths occurred. Univariate analysis showed that the following factors were significantly associated with ICU admission: greater median number of high-risk medical conditions (2 vs.1; p<0.0001), patient age <5 years (p = 0.002), chronic pulmonary disease (p = 0.01), history of prematurity (p = 0.001), congenital heart disease (p = 0.04), dyspnea (p<0.0001), tachypnea (p = 0.003), lower median oxygen saturation (SpO_2_ 92% vs. 98%; p <0.0001), acute renal failure (p = 0.004), and an infiltrate on chest radiograph (p<0.0001). Multivariate analysis showed that dyspnea (p = 0.01) and oxygen saturation (median SpO_2_ 92% vs. 98%; p = 0.02) were significantly associated with ICU admission (Table A2).

## Conclusions

We sought to identify predictors of hospitalization in patients with confirmed pandemic (H1N1) 2009 infection. Univariate analysis showed that presence of high-risk medical conditions, age <5 years, dyspnea, and findings of tachypnea, hypoxia (SpO_2_ <92%), chest radiograph infiltrate, and acute renal failure were significant risk factors for hospitalization. Notably, headache, rhinorrhea, sore throat, and cough were inversely associated with hospital admission. We hypothesize that treating physicians perceive these symptoms as more suggestive of upper respiratory tract disease and hence are less likely to hospitalize such patients.

Multivariate analysis showed that only a higher number of high-risk medical conditions (including age <5 years), dyspnea, and a lower median oxygen saturation level were predictive of hospitalization. We found that dyspnea and a low median oxygen saturation level were also associated with ICU admission. These findings suggest that clinicians’ decision to hospitalize was not influenced by mere perception of illness severity, but rather it accurately reflected the risk for complicated or severe disease. Our findings are also consistent with CDC alerts on emergency warning signs of pandemic (H1N1) 2009 influenza (*8*) and a recent report by Echevarría-Zuno et al. (*9*) from Mexico in which dyspnea, tachypnea, and cyanosis were prognostic factors for admission and death. Although the presence of any 1 underlying high-risk medical condition has been previously described as a risk factor for complication with seasonal influenza including hospitalization (*10*), a higher number of high-risk medical conditions is a stronger predictor of hospitalization (median number 2 vs. 0; p = 0.01).

Although excellent clinical prediction rules for hospitalization of patients with community-acquired pneumonia are available (e.g., CURB-65 or pneumonia severity index), few data exist for influenza admissions (*10*,*11*). We propose a simple clinical guide for hospitalization of patients with pandemic (H1N1) 2009 infection by using results of our multivariate analysis. The presence of any of 3 predictors—>2 high-risk medical conditions (including age <5 years), dyspnea, or hypoxia—has sensitivity, specificity, positive predictive value, and negative predictive value of 72%, 88%, 79%, and 83%, respectively, for hospitalization. Dyspnea or hypoxia was also predictive of ICU admission, with sensitivity, specificity, positive predictive value, and negative predictive value of 94%, 91%, 71%, 98%, respectively. Identification of these risk factors is widely applicable as a triage tool, especially in settings such as physician’s offices where laboratory and radiologic data are not immediately available.

## Appendix

**Table A1:** Multiple logistic regression for risk factors associated with hospitalization for pandemic (H1N1) 2009 (nonhospitalized vs. hospitalized patients)*

Variable	Coefficient (β)	SE	p value	OR (95% CI)
Intercept	81.5	28.8	–	–
No. high-risk conditions†	1.24	0.48	0.01	3.44 (1.34–8.83)
Dyspnea	3.09	1.20	0.01	22.00 (2.12–228.80)
O_2_ saturation	–0.86	0.30	0.004	0.42 (0.24–0.76)

*OR, odds ratio; CI, confidence interval; O_2_ Saturation, oxygen saturation. Variables included in regression analysis: age <5 y, number of high-risk Centers for Disease Control and Prevention conditions, history of prematurity, hemoglobinopathy, or chronic neurologic disease, presence of dyspnea, tachypnea, oxygen saturation, and acute renal failure. Chest radiograph infiltrate was not included in the model because only half of the study received a chest radiograph. This model was well-fitted with a Hosmer-Lemeshow statistic of 0.773. †A high-risk condition as defined by the Centers for Disease Control and Prevention: age <5 y or >65 y; pregnancy; immunosuppression; chronic pulmonary, cardiovascular, hepatic, hematologic, neurologic, neuromuscular, or metabolic disorders; and long-term aspirin therapy in persons <18 y.

**Table A2:** Multiple logistic regression for risk factors associated with ICU admission for pandemic (H1N1) 2009 (ICU patients vs. non-ICU study cohort)*

Variable	Coefficient (β)	SE	p value	OR (95% CI)
Intercept	44.2	20.6	–	–
Dyspnea	3.20	1.29	0.01	24.4 (1.95–305.4)
O_2_ saturation	–0.55	0.24	0.02	0.58 (0.36–0.92)

*ICU, intensive care unit; OR, odds ratio; CI, confidence intervals; O_2_ saturation, oxygen saturation. Variables included in regression analysis: age <5 y, number of high-risk conditions per Centers for Disease Control and Prevention, history of prematurity, congenital heart disease or pulmonary disease, the presence of dyspnea, tachypnea, O_2_ saturation, and acute renal failure. Chest radiograph infiltrate was not included in the model because only half of the study cohort received a chest radiograph. This model was well-fitted with a Hosmer–Lemeshow statistic of 0.923.
